# Anesthesia-Associated Relative Hypovolemia: Mechanisms, Monitoring, and Treatment Considerations

**DOI:** 10.3389/fvets.2018.00053

**Published:** 2018-03-16

**Authors:** Jessica Noel-Morgan, William W. Muir

**Affiliations:** ^1^Center for Cardiovascular & Pulmonary Research, The Research Institute at Nationwide Children’s Hospital, Columbus, OH, United States; ^2^QTest Labs, Columbus, OH, United States; ^3^College of Veterinary Medicine, Lincoln Memorial University, Harrogate, TN, United States

**Keywords:** relative hypovolemia, distributive shock, mean circulatory filling pressure, anesthesia, fluid therapy, functional hemodynamics, dynamic index, preload responsiveness

## Abstract

Although the utility and benefits of anesthesia and analgesia are irrefutable, their practice is not void of risks. Almost all drugs that produce anesthesia endanger cardiovascular stability by producing dose-dependent impairment of cardiac function, vascular reactivity, and compensatory autoregulatory responses. Whereas anesthesia-related depression of cardiac performance and arterial vasodilation are well recognized adverse effects contributing to anesthetic risk, far less emphasis has been placed on effects impacting venous physiology and venous return. The venous circulation, containing about 65–70% of the total blood volume, is a pivotal contributor to stroke volume and cardiac output. Vasodilation, particularly venodilation, is the primary cause of relative hypovolemia produced by anesthetic drugs and is often associated with increased venous compliance, decreased venous return, and reduced response to vasoactive substances. Depending on factors such as patient status and monitoring, a state of relative hypovolemia may remain clinically undetected, with impending consequences owing to impaired oxygen delivery and tissue perfusion. Concurrent processes related to comorbidities, hypothermia, inflammation, trauma, sepsis, or other causes of hemodynamic or metabolic compromise, may further exacerbate the condition. Despite scientific and technological advances, clinical monitoring and treatment of relative hypovolemia still pose relevant challenges to the anesthesiologist. This short perspective seeks to define relative hypovolemia, describe the venous system’s role in supporting normal cardiovascular function, characterize effects of anesthetic drugs on venous physiology, and address current considerations and challenges for monitoring and treatment of relative hypovolemia, with focus on insights for future therapies.

## Introduction

To survive anesthesia is to survive a potentially life-threatening event. What other form of medical practice is designed to intentionally depress or inhibit a spectrum of neurophysiologic processes so as to be able to painlessly inflict varying degrees of medical or surgical psychological or physical trauma. Although the potential benefits and utility of anesthesia and analgesia are obvious, the practice of anesthesia is not without risk, particularly in animals. Indeed, the adverse events associated with anesthetizing animals, although similar to those reported in humans, are far more common than reported for humans (1–4). A recent study investigating adverse events associated with anesthesia in dogs and cats suggested that approximately 40% of animals had at least one adverse event and as many as 1% had up to six adverse events (5). Anesthetic death is reported to occur in approximately 0.5, 1.0, and 10 in every 1,000 anesthetic episodes in otherwise healthy dogs, cats, and horses, respectively (6–10). These rates are two to three orders of magnitude greater than those reported for healthy humans (approximately 0.001 per 1,000) (1). Among the many potential explanations for this discrepancy, human error, inadequate training, lack of experience or familiarity with the drugs and equipment used to produce anesthesia, insufficient monitoring, and haste or distraction, have been identified as specific causes for adverse outcomes in human medicine (3). Species differences aside, the incidence of adverse events, including intraoperative cardiac arrest, is considerably greater in animals than in humans (2, 8, 9, 11). Reemergence from anesthesia, breakthrough pain, hypoventilation, respiratory arrest, airway complications, and hypotension are comparatively common adverse events reported in dogs, cats, and horses (5, 8, 10, 11). Anesthesia-associated hypotension is frequently attributed to a decrease in ventricular contractile performance, arterial vasodilation, or both (5, 10–14). Far less emphasis has been placed upon alterations in venous physiology or the effects of anesthetic drugs on the venous system’s contribution to cardiac output (CO). Increasing evidence, however, suggests that anesthetic drugs produce significant and clinically relevant effects on venous function that result in increases in venous capacitance and compliance, and a reduced response to vasoactive substances (15–17). Anesthetic drug impairment of venous function is an insidious and relatively unappreciated cause of relative hypovolemia that reduces CO, predisposes to hypotension, and can lead to vasodilatory shock especially in sick (e.g., septic), depressed, or debilitated animals (17–19). The focus of this short perspective is to define relative hypovolemia, describe the function of the venous system and its role in maintaining normal cardiovascular function, emphasize the effects of anesthetic drugs on venous physiology, and outline considerations for monitoring and treating relative hypovolemia.

## Venous Physiology and CO

**Figure 1:**
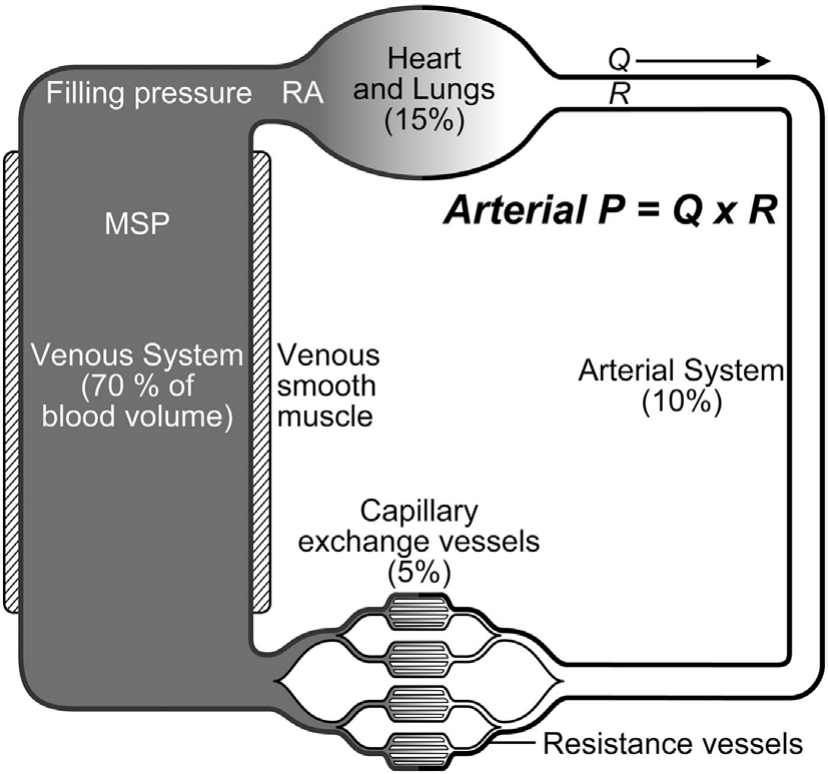
Blood is unevenly distributed throughout the systemic circulation. The large and small veins contain approximately 70% of the blood volume. Arterial pressure (P) is determined by blood flow (Q) and systemic vascular resistance (R); MSP, mean systemic pressure; RA, right atrium.

**Figure 2:**
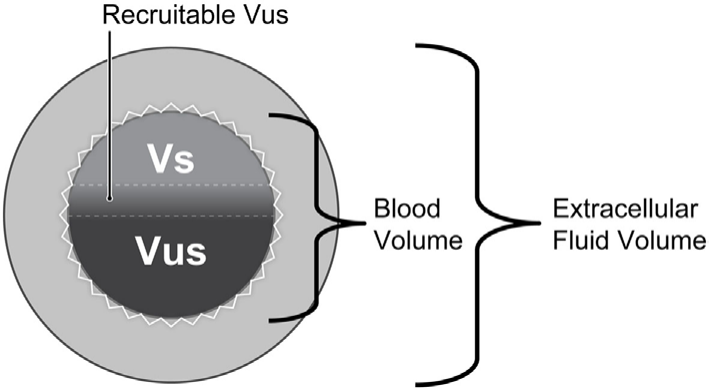
The total blood volume within the vasculature can be divided into two components: stressed circulating blood volume (Vs: approximately 30% of the blood volume) and unstressed intravascular volume (Vus: approximately 70% of the blood volume). The Vs is hemodynamically active (i.e., effective circulating blood volume) and is the principal determinant of the mean systemic pressure, a primary determinant of venous return. The Vus is the volume of blood required to fill the vascular space without increasing blood pressure. A portion of the Vus (up to 15–20 mL/kg; shaded area between the dashed horizontal lines) serves as a blood reservoir and can be recruited to augment the Vs during times of stress or replenish the Vs during hypovolemia. The wavy white line surrounding the inner circle (i.e., volume) suggests that the volume contained therein can become smaller or larger depending upon changes in vasomotor tone.

Maintenance of adequate CO and arterial blood pressure are dependent upon a normal blood volume, vascular tone (arterial and venous), venous return (more appropriately “venous excess”), heart rate (HR), ventricular function, and multiple autoregulatory (compensatory) mechanisms, and are vital for preserving tissue perfusion and oxygen delivery (DO2) (15, 16, 20, 21). Venous return is CO during steady state conditions and is modulated by central venous pressure (CVP): the heart cannot pump what it does not receive. The venous system contains 65–70% of the total blood volume and small veins and venules in the abdomen, spleen, liver, and venous plexus of the skin are more than 30× more compliant than arteries (Figure 1) (15, 22–25). Splanchnic and cutaneous veins contain a large population of both alpha-1 and 2 adrenergic receptors that are highly sensitive to central nervous system sympathetic output, adjustments in baroreceptor reflex activity in response to changes in arterial blood pressure, and endogenous or exogenously administered vasoactive substances (26–29). Splanchnic venous capacitance vessels in particular are much more sensitive to a decrease in carotid sinus pressure or an increase in sympathetic nerve activity than arteries, allowing healthy non-anesthetized animals to lose up to 15–20% of their total blood volume without initiating a significant compensatory hemodynamic response, primarily owing to the reservoir response of the splanchnic veins (26). Alpha-1 adrenergic effects mediated by baroreceptor reflex adjustments contribute significantly to alterations in splanchnic venous capacity (15, 23, 27, 29). Adjustments in venous capacitance aid in maintaining an effective or “stressed” circulating blood volume [the blood volume required to produce measurable increase in transmural pressure: stressed circulating blood volume (Vs)], and are a primary determinant of venous return and therefore CO (15, 16, 21, 24, 30, 31). The unstressed intravascular volume (Vus) is the blood volume required to fill the circulatory system to capacity without increasing cardiovascular transmural pressure (Figure 2) (15, 23). The Vus is composed of a recruitable volume and a residual volume that is functionally analogous to the expiratory reserve and residual volumes that compose the functional residual capacity in the lung. The Vs comprises approximately 30% of the predicted total blood volume (20–25 mL/kg) in most animals, while the Vus can provide a portion of its volume (recruitable reserve volume; approx. 15–20 mL/kg) when maximally activated (24, 28, 31). This volume of blood is equivalent to the administration of 45–60 mL/kg of IV crystalloid, if it is assumed that only one-third of a crystalloid fluid bolus remains in the vascular compartment (32). Only Vs, the “effective” circulating volume, is hemodynamically active, and only a portion of Vus is available to provide a rapidly recruitable reserve volume that can be mobilized during times of need (e.g., exercise, trauma, hemorrhage).

The driving pressure for blood flow returning to the heart from peripheral veins is theorized to be determined by the pressure gradient between a proposed “pivoting pressure,” termed the mean circulatory filling pressure (MCFP), and the right atrium (CVP) (15, 21, 23, 33–35). The MCFP is the equilibration pressure measured at all points in the circulation when the heart is stopped. This pressure is assumed to be located in the venous capacitance vessels, particularly the splanchnic vasculature, and is modified by the effects of both arterial baroreceptor and chemoreceptor reflex mechanisms on venous vascular compliance, and capacitance (36–38). Some consider it to be a flawed and untenable physiologic concept, although many hold the opinion that it does provide a conceptual framework for explaining how changes in venous reservoir compliance and capacitance are associated with alterations in CO when ventricular function is normal or minimally impaired (16, 19–21, 34, 35, 39). Notably, both absolute and relative hypovolemia (decrease in Vs) trigger central and peripheral sympathetically mediated compensatory mechanisms (19, 38). Subsequent activation of alpha-1 receptors in the venous vasculature decreases venous capacitance, aiding in the maintenance of MCFP, venous return and CO by shifting blood from Vus to Vs (24, 27, 40, 41). A compensatory decrease in splanchnic blood volume, for example, has been shown to increase Vs by as much as 10–15 mL/kg in hemorrhaged dogs (25, 28, 31). Importantly, the recruitment (redistribution) of blood from splanchnic and other blood reservoirs (e.g., spleen, lung) may be impaired in animals that are septic, acidotic, hypothermic, aged, or are intolerant of recommended amounts of anesthetic drugs (17, 18).

## Hypovolemia

**Figure 3:**
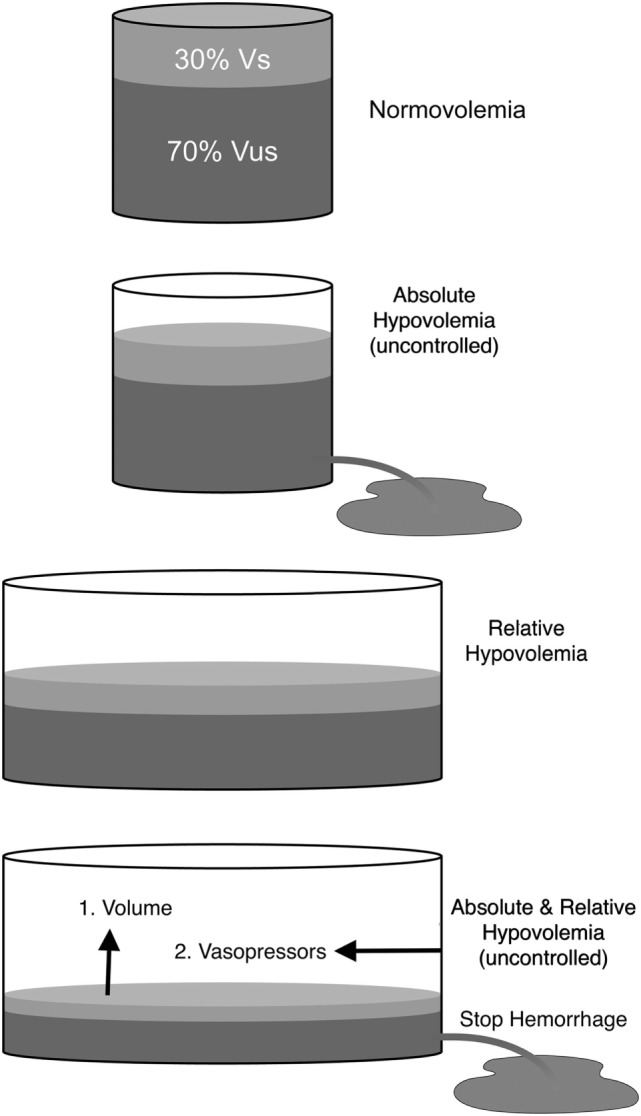
Schematic representation of total blood volume within the vasculature. Normovolemia: blood volume and vascular capacity are normal. Absolute hypovolemia: blood volume is decreased relative to normal vascular capacity (e.g., hemorrhage). Absolute hypovolemia is either uncontrolled (volume loss continues) or controlled (volume loss has been stopped). Relative hypovolemia: blood volume is normal, increased or decreased, but vascular capacity is increased (e.g., anesthetic overdose; sepsis). Absolute hypovolemia is treated with fluids (e.g., crystalloids; blood). Relative hypovolemia is treated primarily with fluids and vasopressors. Vs, stressed circulating blood volume; Vus, unstressed intravascular volume.

Hypovolemia is categorized as either absolute or relative. Absolute hypovolemia (i.e., reduction in total circulating blood volume) is either controlled (hemorrhage that has been stopped) or uncontrolled (hemorrhage that has not stopped) and implies the loss of blood, plasma or water from the vascular compartment. Absolute hypovolemia can be conceptualized as a decrease in blood volume relative to a normally sized vascular compartment (Figure 3). Alternatively, relative hypovolemia implies a normal, or possibly increased, blood volume that is not adequate to fill the vascular compartment because the volume (capacity) of the vascular compartment has increased. Hypovolemia from any cause can reduce venous return, CO and arterial blood pressure, regardless of whether or not compensatory mechanisms are inadequate or impaired, thereby limiting tissue perfusion and DO2 to tissues (15, 16). Severe hypovolemia leads to the development of oxygen debt and is directly correlated with lactic acidemia and mortality (42, 43). Vasodilation, predominantly venodilation, is an important cause of relative hypovolemia produced by anesthetic drugs and can be exacerbated in sick, septic, hypothermic, or aged animals. Relative hypovolemia frequently contributes to low CO and hypotension during anesthesia and is a more frequent, insidious, and occult mechanism responsible for cardiovascular collapse and death than decreases in HR and cardiac function typically emphasized as the primary reasons for anesthesia-related adverse events (10–14, 17).

## Causes of Relative Hypovolemia

The principal cause for relative hypovolemia is vasodilation, especially venodilation. Vasodilation during anesthesia is a natural consequence of (1) drug toxicity (e.g., sensitivity to anesthetic drugs or anesthetic overdose), (2) impairment or loss of compensatory mechanisms, (3) coexisting or induced metabolic (pH < 7.15) or respiratory (PaCO2 > 80 mm Hg) acidosis; or concurrent, (4) traumatic or surgically induced inflammation, (5) sepsis, (6) cardiogenic shock, and (7) hypothermia. Multiple cellular mechanisms have been implicated in the development of vasodilation and vasodilatory shock that include: a decrease in L-type calcium channel ion transport or myofilament sensitivity to calcium, activation of vascular smooth muscle ATP-sensitive potassium channels (K_ATP_ channels), excess production of the inducible form of nitric oxide (iNOS; e.g., sepsis), and deficiency of the hormone vasopressin (Table 1) (18, 44, 45). Tissue ischemia and/or hypoxia increase intracellular hydrogen ion concentration and decrease cellular ATP production activating K_ATP_ channels resulting in smooth muscle hyperpolarization and vasodilation. Sepsis increases the synthesis of nitric oxide due to the increased expression of iNOS synthase and cGMP generation, resulting in vasorelaxation and resistance to vasoactive drugs (e.g., dopamine, norepinephrine, vasopressin). The combined effects of ischemia-induced acidosis and production of vasodilatory prostaglandins (e.g., PGI2, PGE1, synthetic prostacyclin), activation of K_ATP_ channels, and the production of iNOS in animals that are septic or have chronic heart failure, in conjunction with the confounding effects of acidosis and hypothermia, create an ideal environment for vasodilatation, relative hypovolemia and vascular hyporesponsiveness or refractoriness to fluid administration and the administration vasoactive compounds (46–48).

**Table 1:** General and cellular mechanisms responsible for anesthesia-associated relative hypovolemia.

Decreased central sympathetic output
Decreased cardiovascular reflex responses
Decreased baroreceptor reflex activity
Decreased VSM contractile response or sensitivity to:
Neurohumoral and adrenoceptor agonists (e.g., norepinephrine)
Depressed mechanisms regulating VSM cytosolic Ca^2+^
Reduced VSM intracellular Ca^2+^ concentration
Reduced VSM L-type calcium channel ion transport
Reduced VSM myofilament sensitivity to calcium
Activation of K^+^ATP channels

*VSM, vascular smooth muscle; ATP, adenosine triphosphate*.

## Anesthetic Mechanisms Responsible for Relative Hypovolemia

Almost all drugs that produce anesthesia endanger cardiovascular stability by producing dose-dependent impairment of cardiac function, vascular reactivity and compensatory autoregulatory responses (Table 2) (17). Most produce some impairment of ventricular function, vascular tone and inhibit central or peripheral sympathetic ganglionic transmission of barostatic control when administered at clinically relevant doses (49–52). All but one (i.e., ketamine) are known to modify multiple vasoregulatory mechanisms, leading to a differential reduction in vascular contractile responses, redistribution of blood flow, and increases in vascular capacitance primarily by inhibition of sympathetic nervous system activity and depression of adrenergic neurotransmission and baroreceptor reflex sensitivity (49–54). Venodilation is an important component of the vascular vasodilatory effects of both injectable (e.g., propofol) and inhalant (e.g., isoflurane) general anesthetics (Table 2) (47–53, 55). Importantly, most hypnotic (GABA-A agonist) intravenous anesthetics generally exert important vascular actions following bolus IV injections, while volatile anesthetics produce vasodilatory effects at clinically relevant concentrations (44). Therapeutic concentrations of both propofol and isoflurane, for example, have been shown to decrease Vs by increasing venous capacitance, while producing minimal changes in either CO or systemic vascular resistance (50, 53, 56, 57). The maintenance of normal or near-normal CO when either drug is administered has been explained by a decrease in the resistance to venous return and slightly improved stroke volume (SV) due to a decrease in afterload (58). In contrast to propofol and isoflurane, both ketamine and etomidate have been shown to produce minimal effects on venous vascular capacitance in normovolemic humans (59, 60). Ketamine decreased venous capacitance in hypovolemic dogs suggesting that it should be considered the drug of choice for induction to anesthesia and as partial intravenous anesthesia in high risk subjects (60). Summarizing, vascular capacitance modulates CO during hemorrhage and acute volume loading (39). Anesthetic drug-induced increases in venous compliance or capacitance predispose to relative hypovolemia and effectively reduce Vs, CO, and DO2 to tissues, potentially leading to the development of oxygen debt. The combination of relative and absolute hypovolemia during anesthesia and surgery in physiologically compromised animals is particularly troublesome, since some animals may rapidly develop irreversible and refractory shock after the loss of relatively small amounts of blood (5–10 mL/kg) (Figure 2).

**Table 2:** Pharmacologic effects of clinically relevant doses of commonly administered anesthetic drugs.^a^

Drug	HR	Arterial blood pressure	CO	Cardiac contractile force	MSP or MCFP	Vasomotor tone	Baroreceptor reflex activity	Sympathetic nerve activity	Splanchnic venous capacitance	Venous return
Inhalant anesthetic	↑↓±	↓↓	↓↓	↓	↓↓	↓↓	↓↓	↓↓	↑↑	↓↓

Injectable hypnotic Propofol Etomidate Barbiturate Neurosteriod Chloralose	±↓ ± ±↑ ±↓ ↓	↓↓ ↓ ± ↓ ±	↓ ↓ ↓ ↓ ±	↓ ↓ ↓ ±↓ ±	↓ ±↓ ±↓ ±↓ ±	↓ ±↓ ↓ ±↓ ±	↓ ±↓ ↓ ↓ ±	↓ ±↓ ↓ ↓ ±	↑↑ ↑ ↑ ↑ ±	↓↓ ±↓ ↓ ↓ ±

Disociative Ketamine Tiletamine	↑ ↑	↑ ↑	↑± ↑±	↑± ↑±	-- --	-- --	-- --	--↑ --↑	-- --	-- --

Opioid Morphine Hydromorphone Fentanyl	↓ ↓ ↓	--↓ --↓ --↓	--↓ --↓ --↓	--↓ --↓ --↓	-- -- --	-- -- --	--↓ --↓ --↓	--↓ --↓ --↓	-- -- --	--↓ --↓ --↓

Alpha-2 agonist	↓↓	↑→↓	↓↓	--↓	↑→↓	↑→↓	--↓	--	↓→↑	↑→↓

Benzodiazepine Diazepam Midazolam	--	--	--↓	--	--↓	--↓	--	--↓	--↓	--↓

Phenothiazine Acepromazine	±↓	↓	--↓	--↓	↓	↓	↓	↓	↑	↓

Local anesthetics Lidocaine Bupivacaine	±↑ ±↑	↓ ↓	--↓ --↓	--↓ --↓	↓ ↓	↓ ↓	-- --	↓ ↓	↑ ↑	↓ ↓

*^a^Clinically relevant dosages are generally equal to or less than those recommended by the manufacturer. Idealized effects expected from normal, healthy humans and animals; ↑, increase; ↓, decrease; ±, increase or decrease; --, little or no change; ↑→↓, increase followed by decrease; ↓→↑, decrease followed by increase. Data compiled from unpublished data (Muir WW, Del Rio CL, Ueyama Y. The effects of anesthetic drugs on mean circulatory filling pressure in isoflurane anesthetized dogs. (2015). Unpublished manuscript.) and (35, 39, 49, 51–53, 57, 61–78)*.

*HR, heart rate; CO, cardiac output*.

## Considerations on Monitoring and Treatment of Relative Hypovolemia

Identifying and treating relative hypovolemia and tissue hypoperfusion may pose a challenge to the anesthesiologist. During anesthesia, maintenance or prompt reestablishment of appropriate DO2 to all tissues is a main concern (17). Effective circulatory volume, cardiac filling, global, regional and microcirculatory flow, and adequate perfusion pressure are all important elements to consider (79, 80).

Presently, perioperative monitoring is largely based on macrocirculatory variables, which may fail to detect relative hypovolemia (81–85). Indeed, despite the recognition of their importance, bedside determination of absolute volemia, monitoring of venous hemodynamics or of microhemodynamics remain cumbersome at best (15). Sophisticated methods for assessment of systemic vascular compliance and Vs have been proposed, but such techniques are yet to be fully validated, particularly in patients with severely compromised vascular tone or receiving vasoactive drugs (86). Therefore, dynamic assessment of a combination of variables along the hemodynamic circuit is helpful for deciphering ongoing processes.

Selection of monitoring procedures depends on a number of factors involving available technology and resources, the anesthesiologist’s familiarity with each technique, patient status, and the surgical or medical procedure being performed. In this regard, continuous clinical reassessment of patient status and anesthetic depth remain important tools that should be applied to all (87). Adding to this, standard hemodynamic and global perfusion monitoring of HR and rhythm, arterial pressures, pulse oximetry, expired gases including end-tidal carbon dioxide and inhalant anesthetic concentrations, arterial blood gases, and lactate, offer a wealth of information, particularly when monitored and interpreted collectively and trended over time (82, 87–90). Of note, ongoing perfusion and oxygenation deficits may occur even when blood pressure is considered normal, and urinary output has been shown to bear limited relation with blood volume, effective blood flow, or renal function during anesthesia (91–93).

Conceptually, CVP is an easily obtainable surrogate to right atrial pressure, capable of providing insights into the interaction between venous return and cardiac function (94). As a single numerical value, it provides limited information, but when appropriately used and interpreted, within the clinical and interventional context, in combination with static and *dynamic* variables (and especially CO or SV, if available), it may add valuable information about a patient’s condition, particularly when values are outside the normal range, or when extreme, unpredicted, or seemingly paradoxical changes occur, related or not with therapeutic interventions (15, 21, 87, 94–96). An excessively high CVP, for instance, may be indicative of right heart failure, increased pulmonary vascular resistance, or volume overload (87). Still, it has been argued in humans that, while a normal CVP is close to zero and the pressure gradient produced by a normal MCFP 8–10 mmHg promotes venous return, any sufficient increase in CVP and/or fall in MCFP may reduce venous return and SV (96). Indeed, elevated CVP has been associated with impairment of microcirculatory flow and acute kidney injury in critical patients (90, 96, 97). Although not a perfect surrogate for mixed venous oxygen saturation, central venous access offers the possibility of central venous oxygen saturation (ScvO2) attainment, in addition to the determination of venoarterial difference in PCO2 (Pv-aCO2). Combined with plasma lactate levels, ScvO2 and Pv-aCO2 offer important information regarding the patient’s status, enabling inferences regarding CO and the presence of dysoxia, sepsis and/or anemia (87, 98).

Measurement of SV and CO is uncommon in veterinary medicine, and is typically reserved for high-risk or critical patients, particularly those refractory to initial therapy (82, 87). Recent reviews have emphasized the limited research validating each method in the veterinary clinical setting, in addition to possible logistic and cost-related considerations (99–101). While several studies have investigated the use of indicator dilution (e.g., using pulmonary artery catheter thermodilution or lithium dilution CO), and echocardiography-based methods for determining CO in different species, these technologies remain impractical in clinical practice (102–110). Of note, echocardiography/Doppler-derived measurements are less invasive, offer unique information on cardiac structure and function, and may offer good estimation of hemodynamic data, but are largely operator-dependent, requiring specialized training and costly equipment (82, 99, 111, 112).

With the aim of sustaining effective circulatory volume, microcirculatory flow and perfusion, the anesthesiologist must assess the appropriateness of fluid administration for each individual patient (summarized by the mnemonic CIT TAIT: context, indication, targets, timing, amount, infusion strategy, and type of fluid) (113), followed by possible use, timing and choice of alternate or ancillary therapy based on vasoactive (pressors, dilators) and/or inotropic support (80, 83, 87, 92, 93, 96, 113–120).

In the context of decreased effective circulating blood volume related to anesthesia and surgical trauma, fluids are generally proposed as a first line therapy, aiming to increase plasma volume, MCFP and the pressure gradient for venous return (83). However, not all patients respond to fluid administration with an increase SV and/or CO (i.e., fluid or preload responsiveness) (121). Beyond fluid dynamics, anesthetic agents and depth, mechanical ventilation (MV), blood flow distribution, endothelial function, integrity of the glycocalyx, and right and left ventricular status all play critical roles in this response (17, 83, 113, 121–124). The question of how to optimize preload, afterload, and contractility remains haunted by the recognition that: both insufficient and excess fluids may result in perfusion deficits and perioperative morbidity; premature or incorrect employment of pressors may also promote further microcirculatory compromise by hindering adequate flow and DO2; and inotropes should be judiciously employed, with guidelines recommending their use only when monitored cardiac function is accompanied by low CO and signs of hypoperfusion despite preload optimization (80, 82, 83, 85, 92, 93, 96, 125, 126).

Newer evidence and monitoring options for fluid resuscitation suggest that formulas for replacement and maintenance should be reexamined (127). Among many proposed strategies (e.g., “liberal,” “restrictive,” “zero-balance,” “dynamic fluid balance,” and “goal-directed” therapies), a universal algorithm accounting for all possible patient-case combinations remains unrealistic. Current recommendations propose a preplanned approach, tailored to each patient, that employs fluids only on clear indication (83, 92, 93, 113, 125, 127–131). To this end, functional hemodynamics, using *dynamic* indices such as systolic pressure variation (SPV), pulse pressure variation (PPV), stroke volume variation (SVV), plethysmographic variability index, aortic flow peak velocity variation (ΔVpeak), and caudal vena cava distensibility index (CVCDI), have demonstrated promise in predicting preload responsiveness and help guide fluid therapy (82, 83, 92, 121, 132–137).

Comprehensive studies, meta-analyses, and reviews are available that elaborate on use of *dynamic* indices to guide fluid therapy (121, 137–139). Among important highlights, full awareness of all mechanisms and limitations pertaining to each is essential. For instance, many of these methods require MV within very specific settings, and without breathing efforts or arrhythmias during the measurement period (140, 141). Patient cardiovascular and pulmonary status, and particularities of surgical interventions, are also important factors that impact cardiopulmonary interactions and related pressure gradients (121, 132–134, 136, 137, 141–145). Spontaneous breathing, right ventricular (RV) failure, and increased RV afterload have been associated with false-positive results for PPV and SVV (i.e., elevated values not related to preload responsiveness) (95, 146–150). False-negative results have been observed with insufficient tidal volumes (Vt), decreased lung compliance, and increased vascular compliance (95, 141, 144, 151–153). Other conditions possibly altering cutoff values or compromising their effectiveness are elevated positive end-expiratory pressure, increased Vt, changes in vascular tone, increased abdominal pressure, and changes in chest wall compliance (Table 3) (111, 118, 140, 142, 154–159). It is important to note that clinical use of these indices must be investigated in detail, in each species, before translation into clinical practice is feasible. For example, does the dogs’ greater chest wall compliance relative to lung compliance impact predictive and cutoff values for each *dynamic* index (160–162). Lower HR and respiratory rates in horses may also pose limitations (140). Among veterinary-pertinent studies (105, 118, 122, 160–173), a recent investigation with hypotensive dogs found PPV ≥ 15% had 50% sensitivity and 96% specificity in predicting preload responsiveness, further estimating PPV ≥ 19.5 for 100% sensitivity (76% specificity) (171). Another investigation with healthy dogs disclosed cutoff values for ΔVPeak ≥ 9.4% (89% sensitivity, 100% specificity), SPV ≥ 6.7% (78% sensitivity, 93% specificity), and CVCDI ≥ 24% (78% sensitivity, 73% specificity), as being predictive of preload responsiveness (172). These promising results warrant further investigations under different clinical and operative scenarios. A concept to be kept in mind, however, is that, even when potentially preload-responsive, the assessment of whether fluids are actually needed, tolerated, or the best management for the condition requires comprehensive clinical judgment, considering the patient’s pathophysiological status (81, 82, 85, 113, 174, 175). *Dynamic* indices may, nevertheless, offer an additional piece of information to help optimize fluid therapy and further aid decisions targeting use and timing of ancillary or alternate therapeutic interventions (176).

**Table 3:** Factors potentially interfering with PPV and SVV.^a^

Spontaneous breathing
Cardiac arrhythmias
Tidal volume (Vt, insufficient, excessive)
Elevated positive end-expiratory pressure
Inspiratory to expiratory ratio
Heart rate to respiratory rate ratio
Lung compliance
Chest wall compliance (including open chest)
Increased right ventricular afterload
Increased intraabdominal pressure
Right and/or left ventricular failure
Increased vascular compliance
Changes in vascular tone

*^a^Refer text for details*.

*PPV, pulse pressure variation; SVV, stroke volume variation*.

Species-specific clinical trials investigating the efficacy and safety of IV fluid resuscitation are woefully underrepresented in the veterinary literature (83, 87, 124, 174, 177, 178). Those that do exist are frequently poorly designed, uncontrolled, and underpowered (117). Even fewer studies have focused on the volume kinetics of IV fluids for the treatment of relative hypovolemia associated with injectable or inhalant anesthetic protocols. One study in isoflurane (3%) anesthetized dogs demonstrated that 80 mL/kg of a balanced electrolyte solution (Plasmalyte-A) produced no effect on arterial blood pressure, SV or CO until the inhalant anesthetic concentration was reduced to 1.6% (122) suggesting that IV fluid therapy may be useless as a treatment for anesthetic-associated relative hypovolemia. Another investigation in hypotensive isoflurane anesthetized dogs concluded that arterial blood pressure measurements were a poor predictor of the hemodynamic response to fluid administration (179). Current suggested guidelines for dogs, cats, and horses have, for the most part, been adopted from experimental studies in rodents, dogs, and pigs, volume kinetic studies conducted in sheep and humans, and clinical trials or meta-analyses completed in humans (180–200). These experimental and clinical studies suggest that fluid choice, optimal fluid volumes (mL/kg), and the rate of fluid administration (mL/kg/min or h) are context-sensitive (i.e., physical condition, age, surgical procedure, anesthetic choice, etc.) and highly likely to be species-dependent, highlighting the importance of personalizing fluid resuscitation protocols. Taken together these studies suggest that: (1) goal-directed fluid therapy is superior to “rules of thumb” (e.g., 3 mL crystalloid/1 mL blood loss) or standardized formulas (3–10 mL/kg/h); (2) a balanced crystalloid solution (201), is the best first choice fluid unless laboratory data suggest otherwise; (3) monitoring techniques should include at least one validated *dynamic* index [e.g., PPV (165, 170, 171, 173)]; (4) an IV fluid bolus should not exceed 20–30 mL/kg (199); and (5) maximal rates of fluid administration should range from 0.02 (maintenance) to 1.0 mL/kg/min (resuscitation) during anesthesia (200).

Fluid therapy for the treatment of anesthetic-associated relative hypovolemia and hypotension remains largely ineffective and predisposes to fluid overload (87, 202, 203). Not unintentionally, CIT TAIT implies an option to “sit tight” and withhold fluids temporarily or longer (113). In this regard, aside from replenishing Vus reserves, fluid administration should aim to restore Vs and target specified hemodynamic improvement, further considering long term (e.g., impact on organ function, ICU days, survival) measurable outcomes (174, 178, 204). The most appropriate sequence of events to be considered for treating anesthetic-associated hypotension or signs of low blood flow (e.g., prolonged capillary refill time, weak peripheral pulses, increased PPV) during anesthesia should be to: (1) adjust [e.g., stop; reduce, refine, replace: (3R’s of anesthesia)] the anesthetic protocol, (2) administer a balanced crystalloid based upon clinical signs and monitoring data, and (3) administer a vasoactive (e.g., norepinephrine, vasopressin) drug for vasodilation or inotropic (e.g., dobutamine) drug for poor cardiac performance (205). All three events may be required simultaneously, especially in high-risk subjects that have already lost blood (>15–20 mL/kg) or are septic (Figure 3). Notably, the volatile anesthetics sevoflurane and isoflurane have been shown to preserve the endothelial glycocalyx against injury in ketamine anesthetized rats (206–209), whereas propofol increases glycocalyx shedding and vascular permeability (210) and excessive fluid administration triggers atrial natriuretic peptide release increasing vascular membrane permeability and interstitial fluid accumulation (202). Dexmedetomidine has been demonstrated to produce protective effects against ischemia–reperfusion injury in heart, kidney, and brain in rodent animal models (211). These beneficial drug-related actions combined with each drug’s known effects on MCFP (Table 2) suggest that balanced anesthesia with isoflurane, ketamine, and dexmedetomidine may help to limit the development of anesthetic-associated endothelial glycocalyx injury and relative hypovolemia.

## Conclusion

In summary, anesthesia-induced relative hypovolemia remains an underappreciated and often occult cause of poor tissue perfusion. The venous side of the circulation contains the majority of the blood volume and is a pivotal contributor to SV and CO. Vasodilation, particularly venodilation, is a primary cause of relative hypovolemia induced by anesthetic drugs. As with any hypovolemic state, relative hypovolemia may reduce venous return, CO, tissue oxygen delivery, and eventually arterial blood pressure, when compensatory mechanisms are inadequate or impaired. Tissue oxygen debt can lead to significant morbidity and mortality. Conventional, clinical monitoring, and diagnosis of relative hypovolemia during anesthesia relies on subjective clinical and objective macrohemodynamic measurements (e.g., CVP; arterial blood pressure), and global perfusion assessments (e.g., capillary refill time and color, blood gases, lactate). Beyond correction of anesthetic plane, drug choice, and ventilation, therapeutic intervention typically consists of fluid administration, vasoactive and/or inotropic agents, seeking to optimize preload, afterload and cardiac function, with the ultimate goal of maintaining or restoring the effective circulatory volume (Vs), and microcirculatory flow. While many current fluid therapy strategies and fluid monitoring techniques remain under active research and debate, intravenous fluid therapy remains a first line therapy. Intravenous fluid therapy should be personalized and tailored to each patient’s requirements based upon a clear indication, consideration of potential benefits vs. harms, and objective measures for determining its effects. Variability in current practices related to crystalloid or colloid “fluid bolus,” “fluid challenge,” or assessment of “preload responsiveness” including methods for the assessment of “hemodynamic improvement,” in addition to longer term outcomes, preclude comparisons for substantive conclusions (121, 178, 204, 212–216). Recent studies have focused on data for objective characterization of some of these terms, but no consensus has been established (137, 204, 213, 217–219). Continued research is required, specifically focused on veterinary patients (i.e., for each species and in diverse clinical situations) before they can be effectively translated into clinical practice.

## Author Contributions

WM originated the concept for the article. JN-M and WM contributed to drafting and reviewing the manuscript.

## Conflict of Interest Statement

The authors declare that the manuscript was written in the absence of any commercial or financial relationships that could be construed as a potential conflict of interest.

## Abbreviations

CO, cardiac output; CVCDI, caudal vena cava distensibility index; CVP, central venous pressure; ΔVpeak, aortic flow peak velocity variation; DO2, oxygen delivery; HR, heart rate; iNOS, inducible nitric oxide; K_ATP_ channels, ATP-sensitive potassium channels; LiDCO, lithium dilution cardiac output; MCFP, mean circulatory filling pressure; MSP, mean systemic pressure; MV, mechanical ventilation; PAC, pulmonary artery catheter; PPV, pulse pressure variation; Pv-aCO2, venoarterial difference in PCO2; RV, right ventricle; SPV, systolic pressure variation; SV, stroke volume; ScvO2, central venous oxygen saturation; SvO2, mixed venous oxygen saturation; SVV, stroke volume variation; Vs, stressed circulating blood volume; Vt, tidal volume; Vus, unstressed intravascular volume; VVC, venous vascular capacitance.
